# Supplementary material for: Structural basis of substrate recognition and thermal protection by a small heat shock protein
Source: Nat Commun. 2021 May 21;12:3007. doi: 10.1038/s41467-021-23338-y (PMC8140096; doi:10.1038/s41467-021-23338-y)
Supplement: Supplementary file 1 — Supplementary Information [file 41467_2021_23338_MOESM1_ESM.pdf]

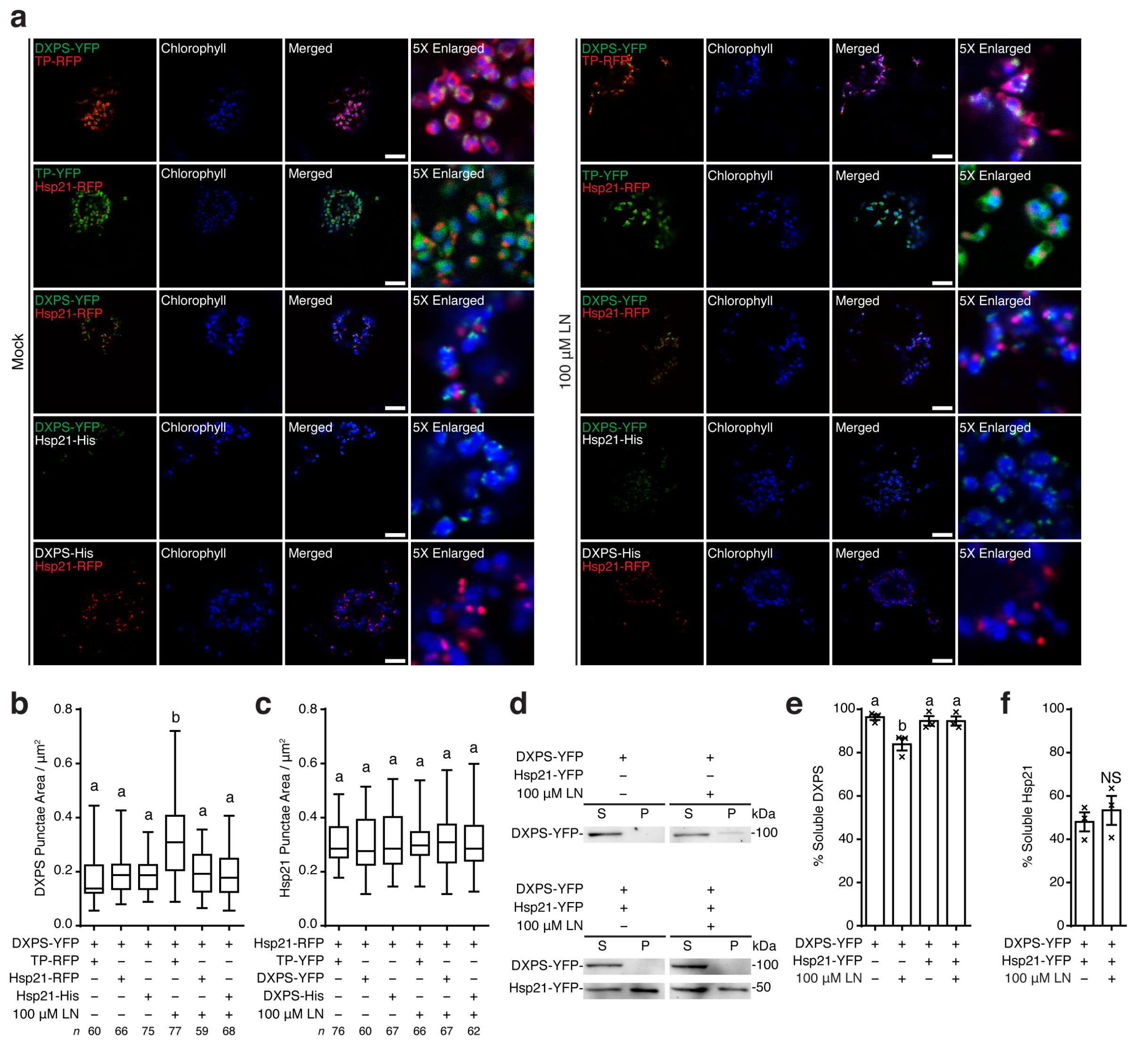

Supplementary Figure 1. DXPS aggregation is resolved by co-expression of Hsp21 *in vivo*. **a,** Confocal microscopy images of chloroplasts in *Arabidopsis* protoplasts (with or without 100 µM LN treatment) expressing DXPS-YFP with TP-RFP, Hsp21-RFP or Hsp21-His, and Hsp21-RFP with TP-YFP or DXPS-His. TP: Rubisco activase RecA transit peptide. Scale bar, 10 µm. **b-c,** Quantification of **b** DXPS punctae area and **c** Hsp21 punctae area as shown in **a**. Values represent mean ± SD, where *n* represented under the graph indicates the number of punctate fluorescent signal. **d,** DXPS and Hsp21 protein distribution in soluble and insoluble fractions isolated from *Arabidopsis* protoplasts with or without 100 µM LN treatment. **e-f,** Densitometry quantification of **e** DXPS and **f** Hsp21 protein distribution in soluble fractions as shown in **d**. Values represent mean ± SEM with $n = 3$ biological replicates. Box and whiskers plots show maxima and minima, upper and lower percentiles (box) and median (line). Letters indicate statistical significance based on one-way ANOVA with post hoc Tukey's multicomparison test ($p \leq 0.05$); means bearing different letters differ significantly. "NS" indicate no statistical significance based on two-tailed unpaired *t*-test. Source data are provided as a Source Data file.

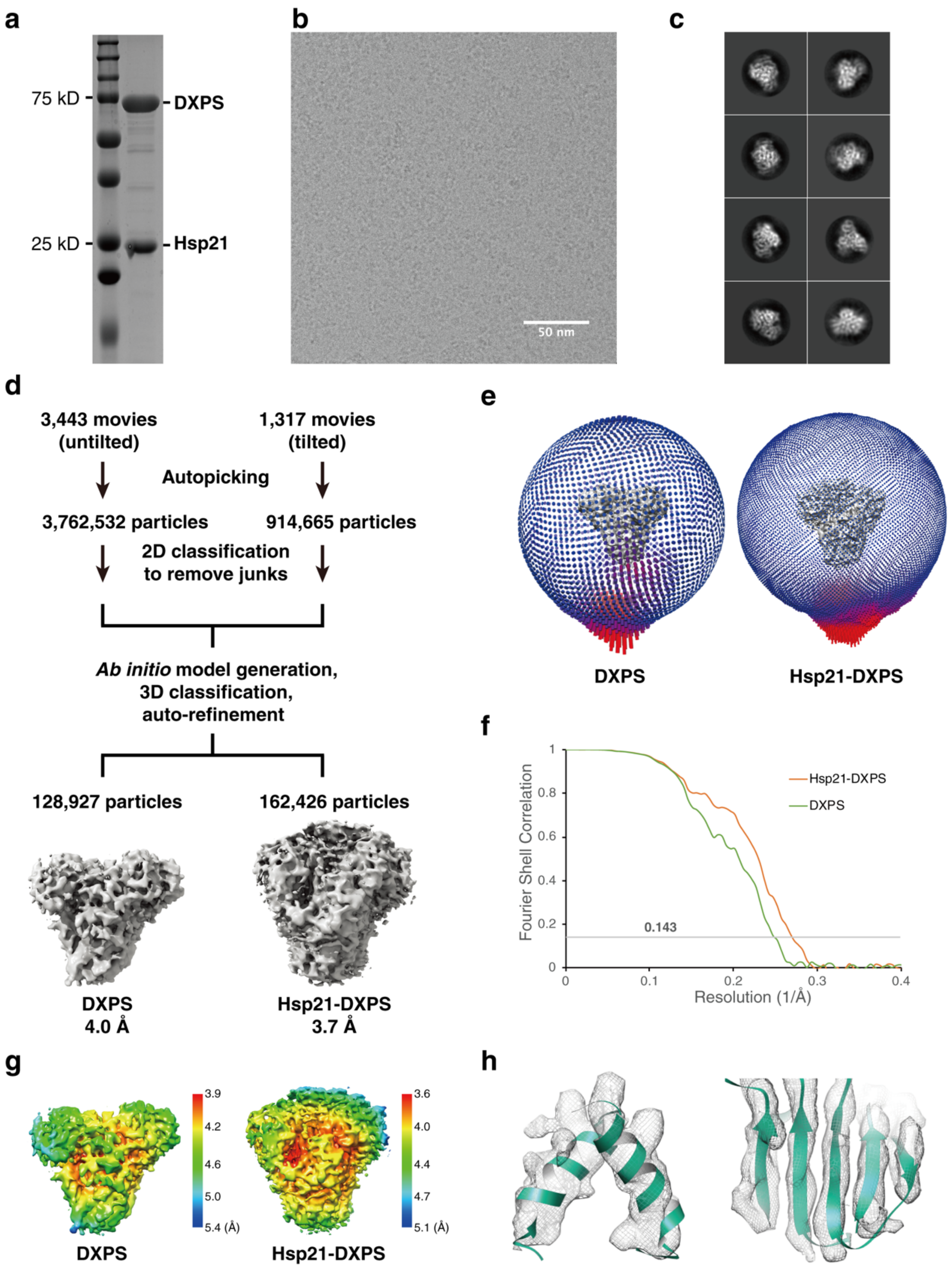

Supplementary Figure 2. Image analysis of Hsp21-DXPS. a, SDS-PAGE gel of the purified Hsp21-DXPS complex. The experiment was repeated at least three times with independent samples and similar results. **b,** A representative cryo-EM micrograph of the Hsp21-DXPS complex out of 4760 movies. Scale bar, 50 nm. **c,** Representative 2D class averages calculated by RELION 3.0. **d,** Summary of the image processing workflow in RELION 3.0. **e,** Euler angle distribution of the particles used in the final refinement. **f,** Fourier Shell Correlation curves following map refinement with the FSC=0.143 threshold indicated. **g,** Local resolution

estimation of the DXPS (left) and Hsp21-DXPS (right) maps analyzed by RELION 3.0. **h,** Selected regions from the Hsp21-DXPS map. Left, bulky side chains are visible in the map density. Right, individual β strands in domain III are well separated. Source data are provided as a Source Data file.

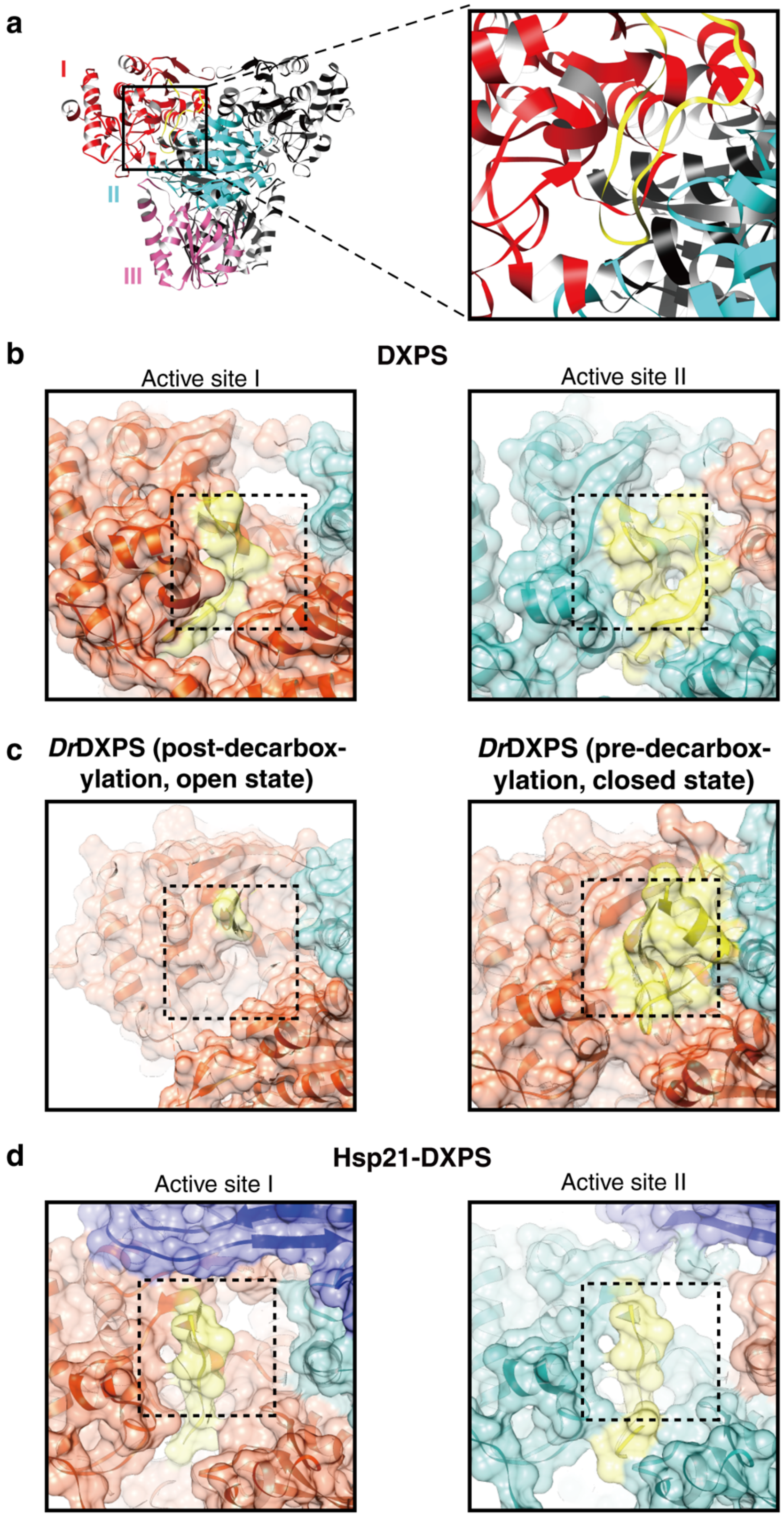

Supplementary Figure 3. Active site conformation of DXPS. a, Opening and closing of the active site located at the interface between domains I and II is regulated by the disorder-to-order transition of part of the gating segment (residues 247-274 shown in yellow). The three

domains of one subunit are colored separately. Location of the active site on one subunit of the DXPS dimer is indicated with a square box with the corresponding zoomed-in view. **b,** The open (Active site I) and the closed (Active site II) conformations of DXPS. **c,** Comparison to the corresponding active site conformations in **b** in the *Dr*DXPS crystal structures (left, PDB 6OUW; right, PDB 6OUV) where the open and the closed sites represent the post- and pre-decarboxylation states of *Dr*DXPS, respectively. **d,** The open conformations of both active sites of DXPS within the Hsp21-DXPS complex are shown. The active sites are indicated with dash boxes.

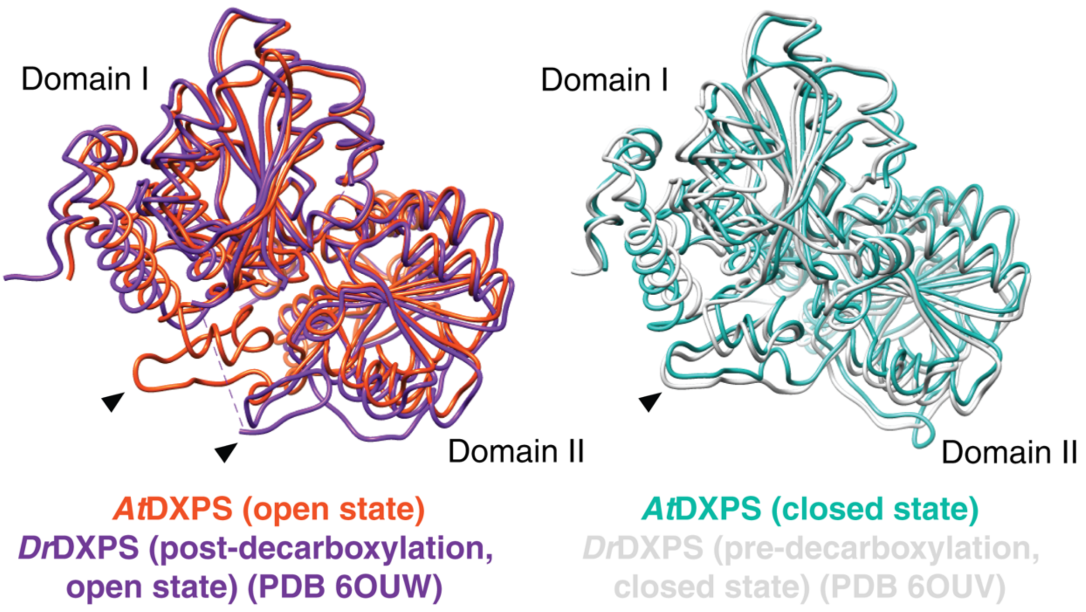

Supplementary Figure 4. Conformation of the linker region of DXPS. The linker regions are indicated by black arrows. Left, The model of *Arabidopsis thaliana* DXPS (*At*DXPS) subunit in the open conformation (orange) is superimposed with the crystal structure of *Deinococcus radiodurans* DXPS (*Dr*DXPS) in the post-decarboxylation, open conformation (purple). Note the difference in the conformations of the linker regions between *At*DXPS and *Dr*DXPS subunits, despite both adopting the open states. The dashed line indicates the missing linker densities in the crystal structure. Right, The model of DXPS subunit in the closed conformation (sea green) is superimposed with the crystal structure of *Dr*DXPS in the pre-decarboxylation, closed conformation (light gray).

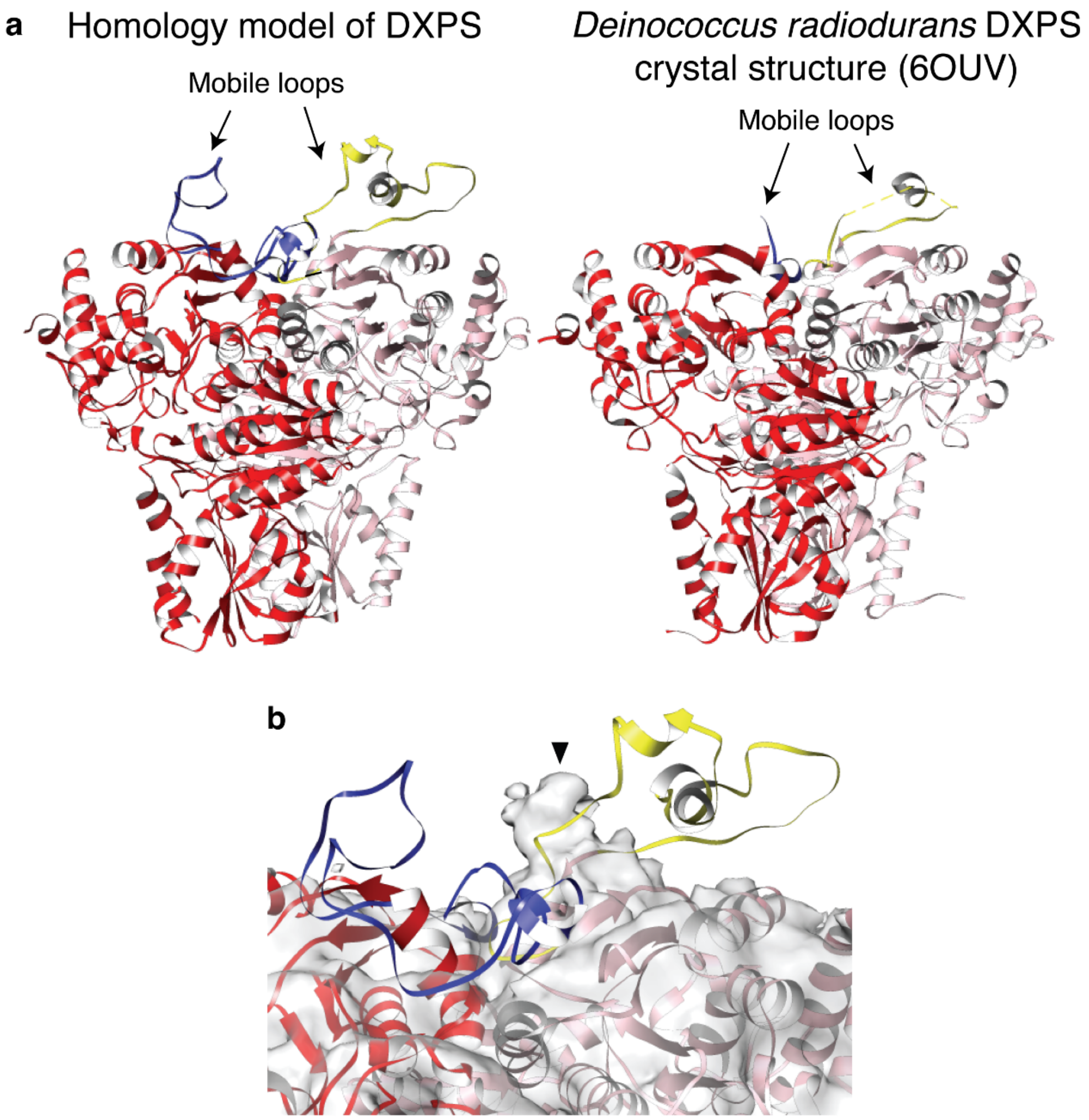

Supplementary Figure 5. The mobile loops of DXPS. a, Homology model of *Arabidopsis thaliana* DXPS generated by the SWISS-MODEL server prior to refinement into the cryo-EM map density (left) and the crystal structure of *Deinococcus radiodurans* DXPS crystal structure (PDB 6OUV) (right). The mobile loops are labeled for each enzyme. The electron density for the mobile loops on the *Dr*DXPS crystal structure are largely missing due to flexibility. **b,** Some densities derived from the mobile loops are only visible (black arrow) in the DXPS cryo-EM map when rendered at low map threshold.

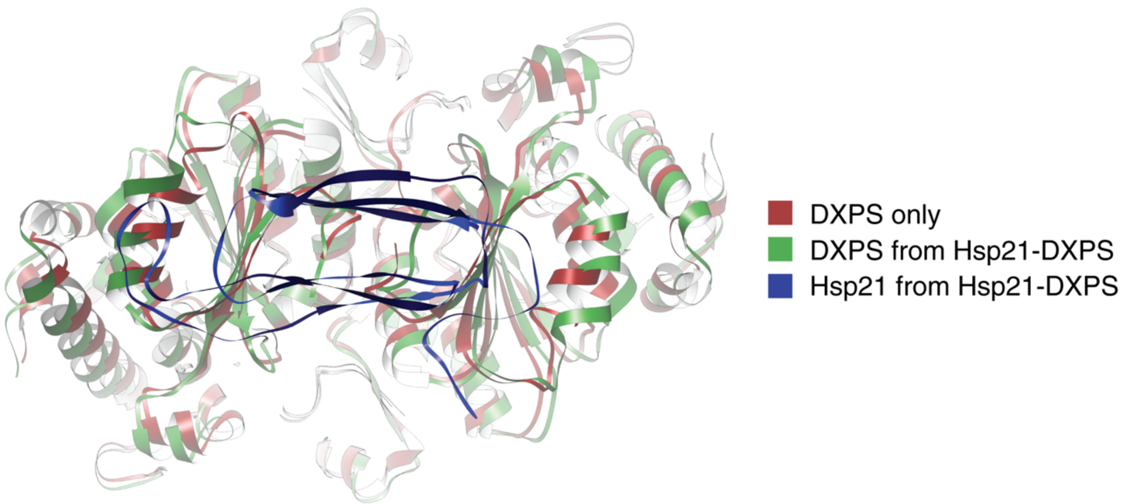

Supplementary Figure 6. Structural changes in DXPS upon Hsp21 binding. Superimposition of the models of DXPS (dark red) and Hsp21-DXPS (green) built from the cryo-EM maps, viewed from the top of the enzyme. The bound Hsp21 in the Hsp21-DXPS complex is shown in blue to show the viewing direction. The Cα RMSD between DXPS models is 1.76 Å.

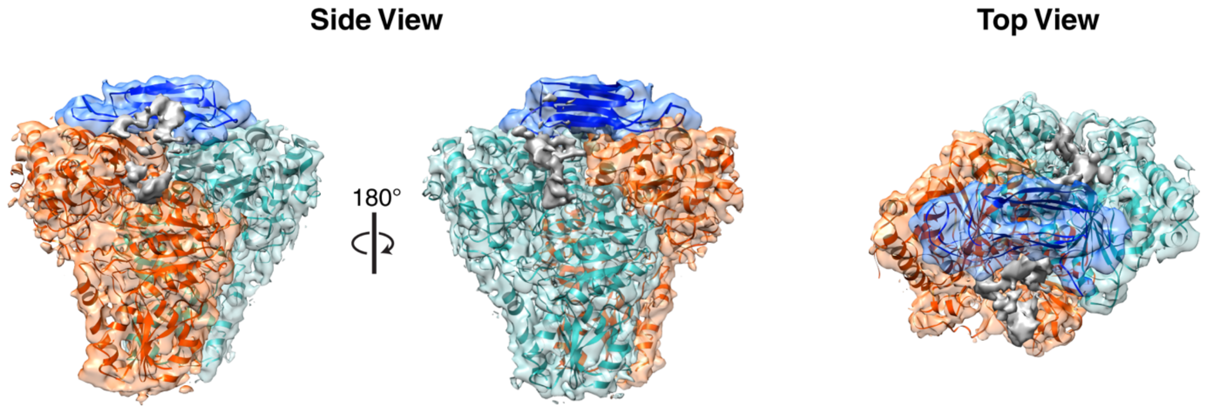

Supplementary Figure 7. Unassigned map densities in the Hsp21-DXPS cryo-EM reconstruction. The unassigned map densities (gray) are overlaid with composite cryo-EM map and the fitted model of Hsp21-DXPS shown in three different views. Located near the interface between Hsp21 and DXPS, these densities are likely contributed by the partially ordered mobile loops of DXPS.

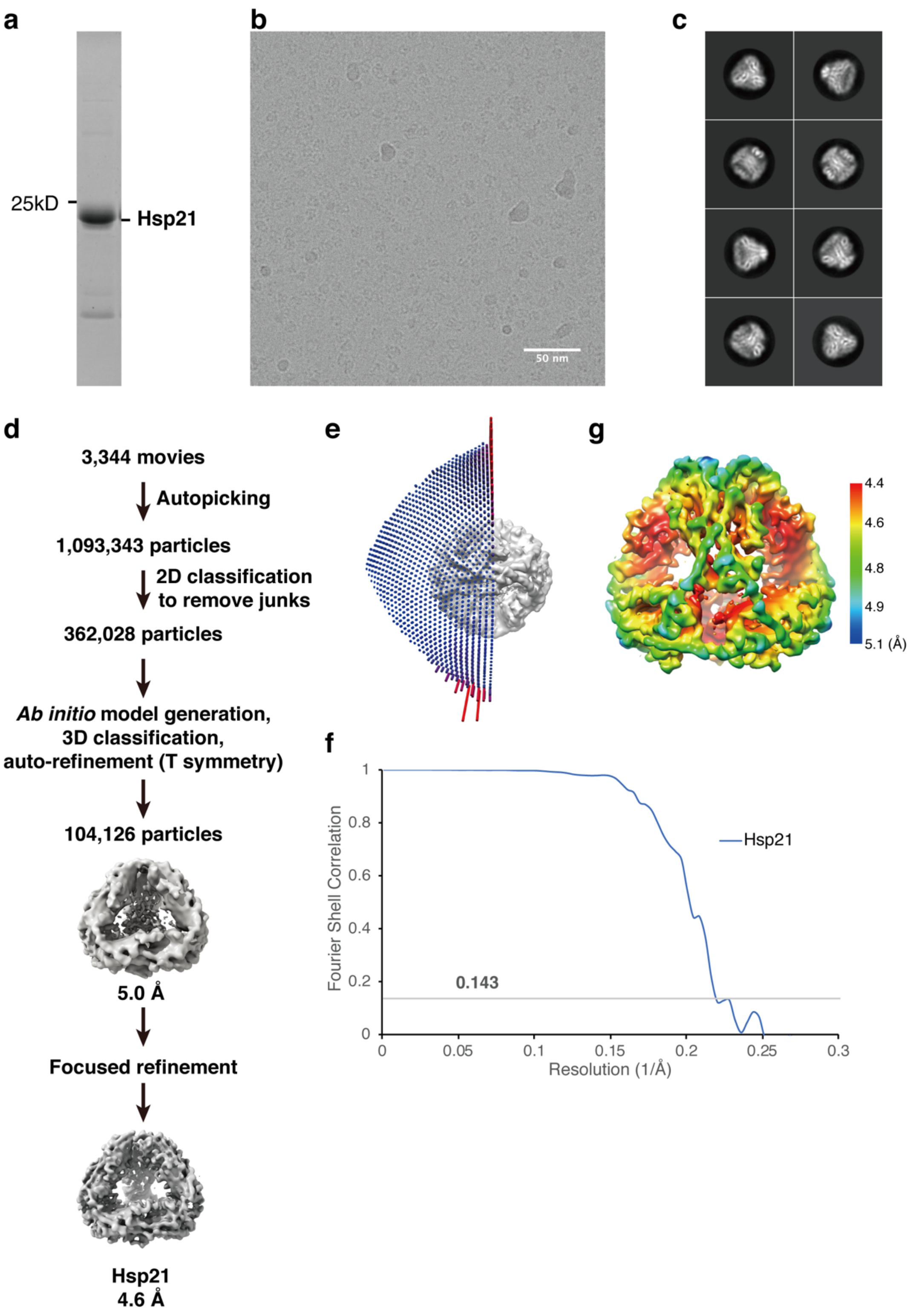

Supplementary Figure 8. Image analysis of Hsp21 dodecamer. a, SDS-PAGE gel of the purified N-terminally His-tagged Hsp21. The experiment was repeated at least three times with independent samples and similar results. **b,** A representative cryo-EM micrograph of His-Hsp21 dodecamer out of 3344 movies. Scale bar, 50 nm. **c,** Representative 2D class averages calculated from RELION 3.0. **d,** Summary of the image processing workflow in RELION 3.0.

Focused refinement by masking out the scattered densities in the inner cavity of the tetrahedron improved the map resolution from 5.0 Å to 4.6 Å. **e,** Euler angle distribution of the particles used in the final refinement. **f,** Fourier Shell Correlation curve following map refinement with the FSC = 0.143 threshold indicated. **g,** Local resolution estimation analyzed by RELION 3.0. Source data are provided as a Source Data file.

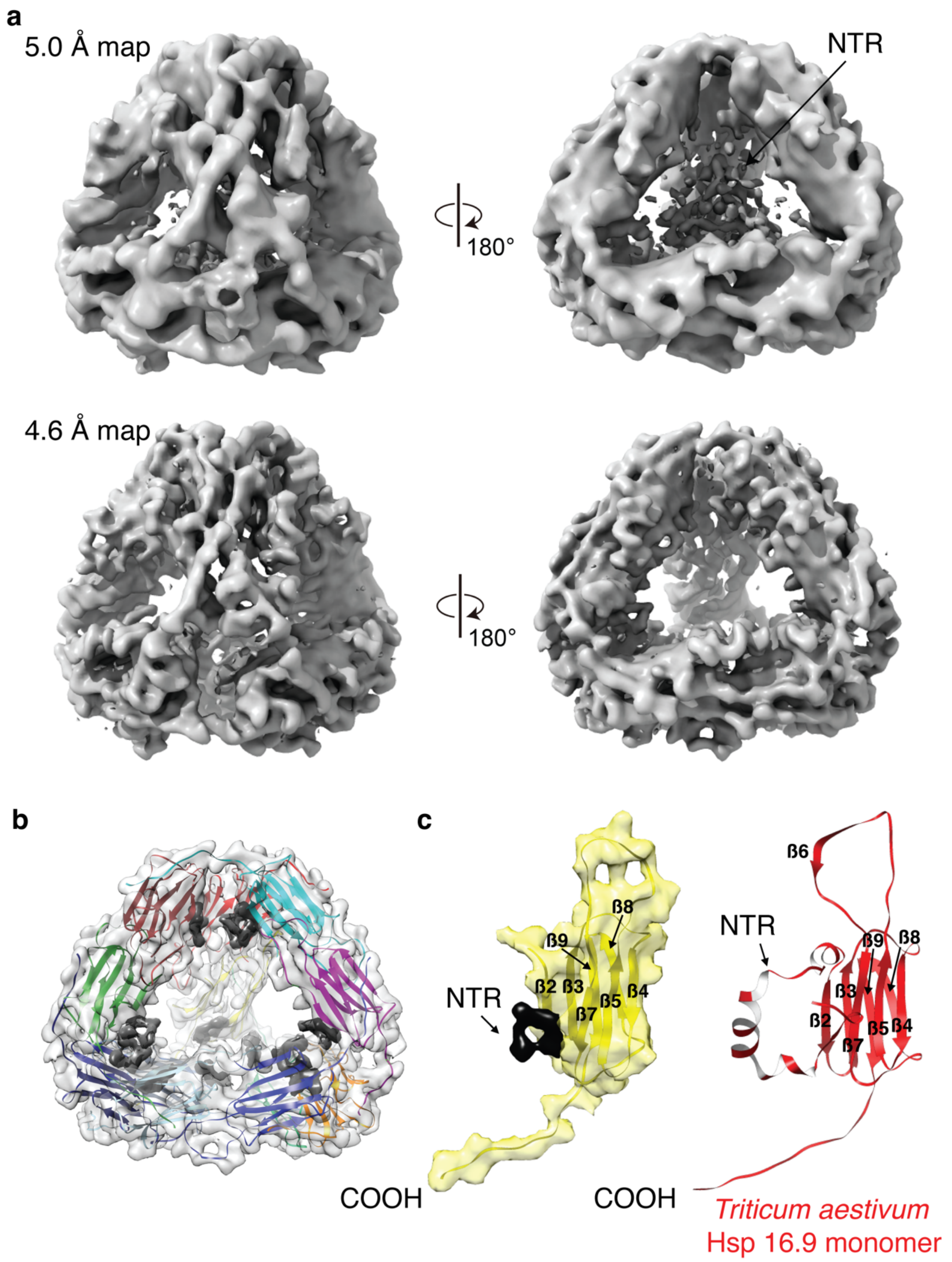

Supplementary Figure 9. The NTR densities in the Hsp21 dodecamer cryo-EM map. a, The 5 Å map of the His-Hsp21 dodecamer prior to focused refinement. The observed scattered densities in the inner cavity of the tetrahedron that are attributed to the NTRs are indicated with an arrow. The 4.6 Å map of the Hsp21 dodecamer is shown for comparison after focused refinement with a soft mask that removed the scattered NTR densities. **b,** The 4.6 Å map fitted with the model. The unassigned residual densities derived from small segments of the NTR are shown in black. **c,** Left, Enlarged view of the Hsp21 monomer. The NTR density

interacting with β7 is shown. Right, Enlarged view of *Triticum aestivum* Hsp16.9 monomer from the crystal structure (PDB 1GME). The distal end of the ordered NTR interacting with β7 is shown for comparison. Note that Hsp16.9 possesses an additional β6 found within the β5-β7 loop.

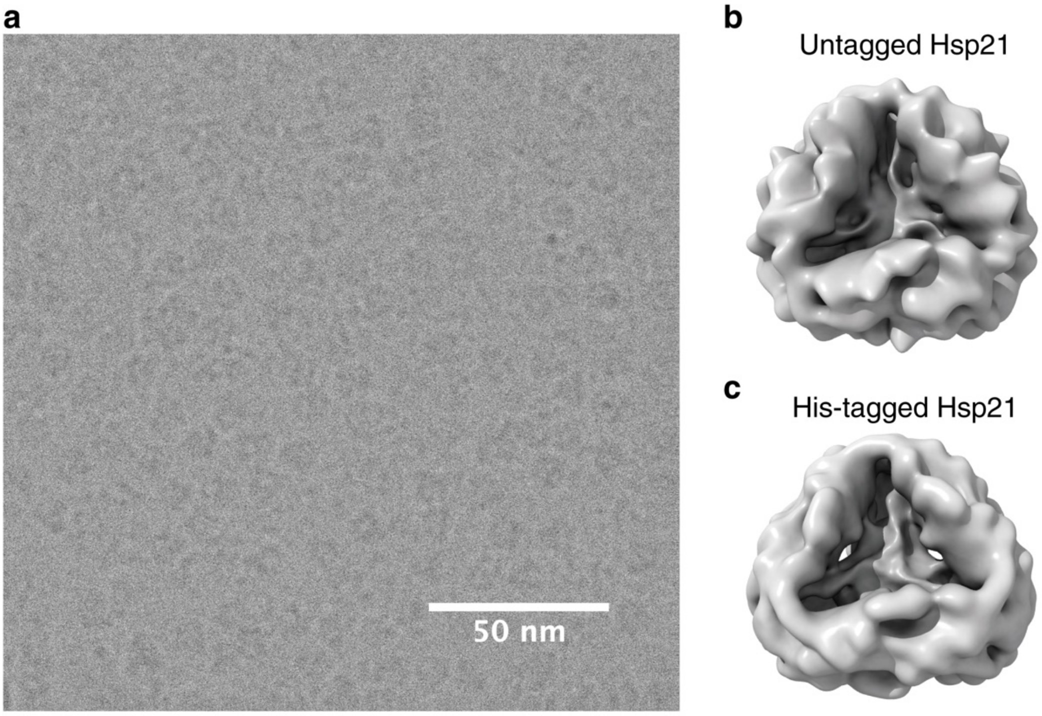

Supplementary Figure 10. The overall architecture of Hsp21 purified without an affinity tag. a, A representative cryo-EM micrograph of the untagged Hsp21 (out of 1022 movies) imaged on a Talos F200C equipped with a Falcon III. Scale bar, 50 nm. **b,** Cryo-EM map obtained from initial refinement of untagged Hsp21 particle images (left) showing the same tetrahedral architecture as the N-terminally His-tagged Hsp21 dodecamer map (right). Both maps are shown as unsharpened map densities. The densities inside in the cavities of both maps are derived from the NTRs.

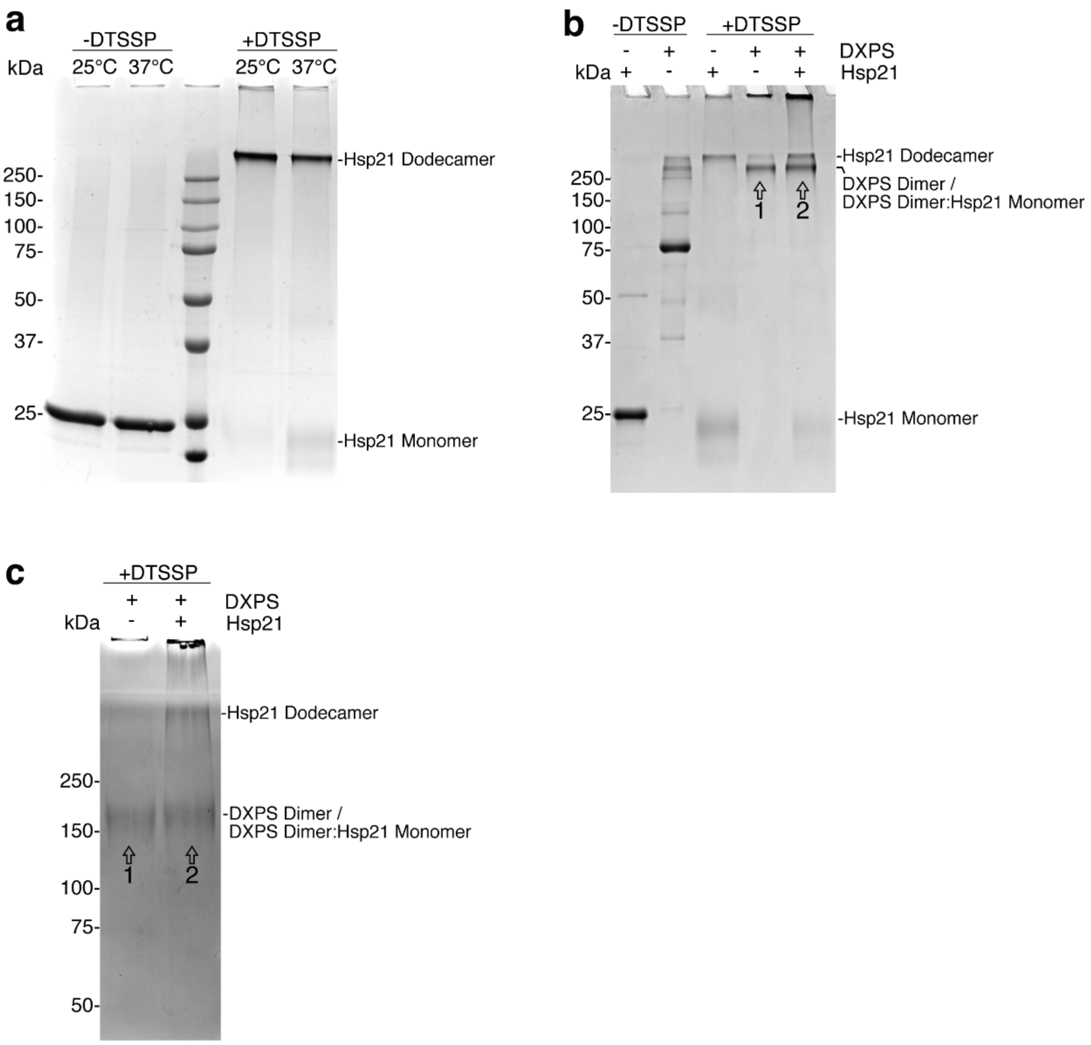

Supplementary Figure 11. Chemical crosslinking between Hps21 and DXPS with DTSSP. **a,** Purified untagged Hsp21 dodecamer was preincubated at 25 °C or 37 °C for 2.5 h, followed by addition of the chemical crosslinker DTSSP (3 mM) for 20 min. **b,** Purified untagged Hsp21 dodecamer and DXPS-Flag in molar ratio of 1:1 were preincubated at 37 °C for 2.5 h, followed by addition of DTSSP (3 mM) for 20 min. Reactions were quenched with 50 mM Tris and samples were analyzed by SDS-PAGE gel. The gel band 1 and 2 indicated in **b** were excised for mass spectrometry analysis to confirm the absence and presence of Hsp21 in the crosslinked products (refer to Supplementary Table 1). Note that the crosslinked DXPS dimer migrates to a position of the gel corresponding to a size that is higher than its expected molecular mass. **c,** The crosslinked DXPS and Hsp21-DXPS proteins were run on a 6 % SDS-PAGE gel. The crosslinked products could not be resolved likely due to the smearing of the gel bands, the small difference between the molecular mass of these two species, and/or crosslinking impacting the migration behavior of these species in the gel. All experiments were repeated at least three times with independent samples and similar results. Source data are provided as a Source Data file.

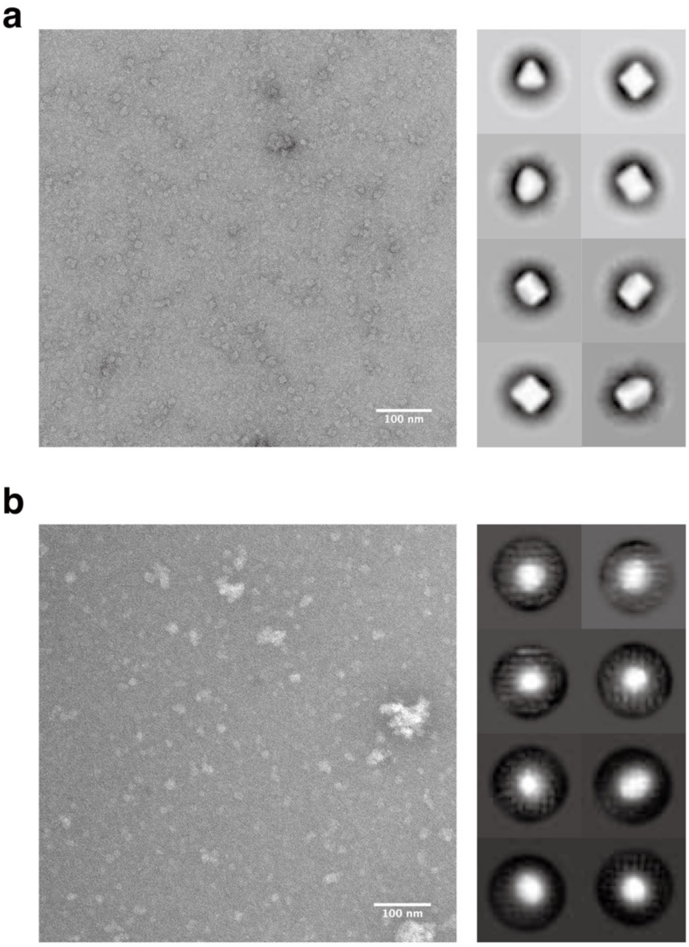

Supplementary Figure 12. Negative stain EM analysis of Hsp21 dodecamer without and with heat treatment. Representative negative stain EM micrographs of purified His-Hsp21 dodecamer **a** without and **b** with heat treatment (1 h at 37 °C) and the corresponding 2D class averages. In total, 151 and 200 micrographs were obtained for Hsp21 dodecamer without and with heat treatment. Scale bar, 100 nm. The class averages showing the structural perturbation of the Hsp21 dodecamer induced by heat treatment is consistent with the proposed dodecamer-monomer dissociation of Hsp21 in response to increasing temperature. Note that Hsp21 monomer could not be directly observed by negative EM due to its small size.

Supplementary Table 1. Mass spectrometry analysis of DXPS and Hsp21-DXPS proteins crosslinked with DTSSP.

No (a)	Accession	Description (Mr/pI(b))	Covered protein %	Matched peptide #	Matched peptide sequence
1	Q38854	1-deoxy-D-xylulose-5-phosphate synthase, chloroplastic (76784/7.04)	2% (19/717AA's)	2	R.GLNVTVADAR.F<br>K.WRPMVLPDR.Y
2	Q38854	1-deoxy-D-xylulose-5-phosphate synthase, chloroplastic (76784/7.04)	7% (51/717AA's)	3	K.QVSLPTATLDGPSPPVGALSSALSR.L<br>R.AYDQVVHDVDLQKLPVR.F<br>K.WRPMVLPDR.Y
2	P31170	Heat shock protein 21, chloroplastic (25328/8.48)	12% (29/227AA's)	2	R.LTMDVSPFGLLDPLSPMR.T<br>R.MFEDTMPVSGR.N

(a) Numbers correspond to the bands marked on Supplementary Fig 11.
(b) Theoretical MW (Da) and pI values.

Supplementary Table 2. Primers used in this study. Underscored portions indicate restriction enzyme sites.

Primer Name	Sequence (5'→3')	Restriction Site
Hsp21-forward (N-terminal hexahistidine tag version)	AACAGAATTCGGAGAACCTGTACTTCCAATCCGCTCAAGACCAGAGAGAAAAC	EcoRI
Hsp21-reverse (N-terminal hexahistidine tag version)	AACAAAGCTTCTACTGAATCTGGACATCGATGACTTTG	HindIII
Hsp21-forward (No tag version)	CGGGGTACCGCTCAAGACCAGAGAGAAAACTCC	KpnI
Hsp21-reverse(No tag version)	CCTTAATTAACTACTGAATCTGGACATCGATGAC	PacI
DXPS-forward (C-terminal hexahistidine-Flag version)	CGGGGTACCGCTTCACTTGCAGAGAAGGG	KpnI
DXPS-reverse(C-terminal hexahistidine-Flag version)	GGCCTTAATTAATCACTTGTCGTCATCGTCTTTGTAGTCGTGATGGTGATGGTGATGAAACAGAGCTTCCCTTGG	PacI
DXPS-reverse (C-terminal Flag version)	CCTTAATTAATCACTTGTCGTCATCGTCTTTGTAGTCAAACAGAGCTTCCCTTGGTG	PacI
DXPS KpnI F	GGGGGTACCATGGCTTCTTCTGCATTTGCTTTTCC	KpnI
DXPS SpeI Non-STOP R	GGGACTAGTAAACAGAGCTTCCCTTGGTGC	SpeI
HSP21 KpnI F	GGGGGTACCATGGCTTCTACACTCTCATTTGCTGC	KpnI
HSP21 SpeI Non-STOP R	GGGACTAGTCTGAATCTGGACATCGATGACTTTGC	SpeI
DXPS KpnI F	GGGGGTACCATGGCTTCTTCTGCATTTGCTTTTCC	KpnI
DXPS-His SacI R	CCCGAGCTCTTAGTGGTGATGGTGATGATGAAACAGAGCTTCCCTTGGTGC	SacI
HSP21 KpnI F	GGGGGTACCATGGCTTCTACACTCTCATTTGCTGC	KpnI
HSP21-His SpeI R	GGGACTAGTTTAGTGGTGATGGTGATGATGCTGAATCTGGACATCGATGACTTTGC	SpeI

Supplementary Table 3. Cryo-EM data collection, refinement and validation statistics.

	**Hsp21-DXPS (EMDB-30263) (PDB 7BZY)***	**DXPS (EMDB-30262) (PDB 7BZX)***	**Hsp21 (EMDB-30261) (PDB 7BZW)**
**Data collection and processing**			
Magnification	75000	75000	75000
Voltage (kV)	300	300	300
Electron exposure (e–/Å^2)	45	45	45
Defocus range (μm)	1.5-2.5	1.5-2.5	1.5-2.5
Pixel size (Å)	1.03	1.03	1.06
Symmetry imposed	C1	C1	T
Initial particle images (no.)	4677197	4677197	1093343
Final particle images (no.)	162426	128927	104126
Map resolution (Å)	3.7	4.0	4.6
FSC threshold	0.143	0.143	0.143
Map resolution range (Å)	3.6-5.1	3.9-5.4	4.4-5.1
**Refinement**			
Initial model used (PDB code)	Homology modeling	Homology modeling	Homology modeling
Map sharpening *B* factor (Å^2)	-203	-216	-329
Model composition			
Non-hydrogen atoms	6169	5633	9756
Protein residues	1260	1151	1212
Ligands	0	0	0
R.m.s. deviations			
Bond lengths (Å)	0.006	0.021	0.003
Bond angles (°)	1.264	2.386	0.817
Validation			
MolProbity score	2.23	1.50	2.66
Clashscore	11.23	2.40	27.81
Poor rotamers (%)	0	0	0
Ramachandran plot			
Favored (%)	84.64	92.30	80.64
Allowed (%)	15.04	6.21	19.36
Disallowed (%)	0.32	1.49	0.0

*: Poly-alanine
