# Supplementary material for: Structural basis of substrate recognition and thermal protection by a small heat shock protein
Source: Nat Commun. 2021 May 21;12:3007. doi: 10.1038/s41467-021-23338-y (PMC8140096; doi:10.1038/s41467-021-23338-y)
Supplement: Supplementary file 3 — Reporting Summary [file 41467_2021_23338_MOESM3_ESM.pdf]

nature research

Corresponding author(s): Wilson Lau Chun Yu

Last updated by author(s): Apr 14, 2021

Reporting Summary

Nature Research wishes to improve the reproducibility of the work that we publish. This form provides structure for consistency and transparency in reporting. For further information on Nature Research policies, see our Editorial Policies and the Editorial Policy Checklist.

Statistics

For all statistical analyses, confirm that the following items are present in the figure legend, table legend, main text, or Methods section.

n/a	Confirmed	
☐	☒	The exact sample size (*n*) for each experimental group/condition, given as a discrete number and unit of measurement
☐	☒	A statement on whether measurements were taken from distinct samples or whether the same sample was measured repeatedly
☐	☒	The statistical test(s) used AND whether they are one- or two-sided<br>*Only common tests should be described solely by name; describe more complex techniques in the Methods section.*
☒	☐	A description of all covariates tested
☐	☒	A description of any assumptions or corrections, such as tests of normality and adjustment for multiple comparisons
☐	☒	A full description of the statistical parameters including central tendency (e.g. means) or other basic estimates (e.g. regression coefficient) AND variation (e.g. standard deviation) or associated estimates of uncertainty (e.g. confidence intervals)
☐	☒	For null hypothesis testing, the test statistic (e.g. F, t, r) with confidence intervals, effect sizes, degrees of freedom and P value noted<br>*Give P values as exact values whenever suitable.*
☒	☐	For Bayesian analysis, information on the choice of priors and Markov chain Monte Carlo settings
☒	☐	For hierarchical and complex designs, identification of the appropriate level for tests and full reporting of outcomes
☒	☐	Estimates of effect sizes (e.g. Cohen's *d*, Pearson's *r*), indicating how they were calculated

Our web collection on statistics for biologists contains articles on many of the points above.

Software and code

Policy information about availability of computer code

Data collection	EPU (Thermo Fisher Scientific), Leica Application Suite X 3.5.5.19976
Data analysis	RELION 3.0, MotionCor2, gCTF 1.06, LOCSCALE , SWISS-MODEL, COOT 0.9, iMODFIT 1.51, PHENIX 1.19.2, MolProbity, Chimera 1.13, ChimeraX 0.8, GraphPad Prism 6, ImageJ 1.53a

For manuscripts utilizing custom algorithms or software that are central to the research but not yet described in published literature, software must be made available to editors and reviewers. We strongly encourage code deposition in a community repository (e.g. GitHub). See the Nature Research guidelines for submitting code & software for further information.

Data

Policy information about availability of data

All manuscripts must include a data availability statement. This statement should provide the following information, where applicable:

- Accession codes, unique identifiers, or web links for publicly available datasets
- A list of figures that have associated raw data
- A description of any restrictions on data availability

Cryo-EM maps are deposited in the Electron Microscopy Data Bank under accession numbers EMD-30261, 30262, 36263. Atomic models are deposited in the Protein Data Bank under accession numbers 7BZW, 7BZX, 7BZY. All other relevant data are available upon request from the corresponding authors. Source data are provided with this paper.

Field-specific reporting

Please select the one below that is the best fit for your research. If you are not sure, read the appropriate sections before making your selection.

☒ Life sciences ☐ Behavioural & social sciences ☐ Ecological, evolutionary & environmental sciences

For a reference copy of the document with all sections, see nature.com/documents/nr-reporting-summary-flat.pdf

Life sciences study design

All studies must disclose on these points even when the disclosure is negative.

Sample size	No statistical method was used to predetermine the sample sizes. Sample size for each experiment is provided in the legends and is mainly based on our past experience performing similar experiments.
Data exclusions	No data were excluded from the analysis.
Replication	Experiments were replicated for multiple times as indicated in the figure legends and method.
Randomization	Randomization is not applicable as there is no group allocation in this study.
Blinding	Blinding is not relevant for structure determination and in vitro studies since the results are not subjective.

Reporting for specific materials, systems and methods

We require information from authors about some types of materials, experimental systems and methods used in many studies. Here, indicate whether each material, system or method listed is relevant to your study. If you are not sure if a list item applies to your research, read the appropriate section before selecting a response.

Materials & experimental systems

n/a	Involved in the study	
☐	☒	Antibodies
☒	☐	Eukaryotic cell lines
☒	☐	Palaeontology and archaeology
☒	☐	Animals and other organisms
☒	☐	Human research participants
☒	☐	Clinical data
☒	☐	Dual use research of concern

Methods

n/a	Involved in the study	
☒	☐	ChIP-seq
☒	☐	Flow cytometry
☒	☐	MRI-based neuroimaging

Antibodies

Antibodies used	Anti-His (Cell Signaling; 27E8), Anti-GFP (Clontech; JL-8), anti-RFP (ChromTek; 5F8)
Validation	https://www.cellsignal.com/products/primary-antibodies/his-tag-27e8-mouse-mab/2366<br>https://www.takarabio.com/products/antibodies-and-elisa/fluorescent-protein-antibodies/green-fluorescent-protein-antibodies?catalog=632569<br>https://www.chromotek.com/products/detail/product-detail/rfp-antibody-5f8/
